# Deep Learning Techniques for Fatty Liver Using Multi-View Ultrasound Images Scanned by Different Scanners: Development and Validation Study

**DOI:** 10.2196/30066

**Published:** 2021-11-18

**Authors:** Taewoo Kim, Dong Hyun Lee, Eun-Kee Park, Sanghun Choi

**Affiliations:** 1 School of Mechanical Engineering Kyungpook National University Daegu Republic of Korea; 2 Division of Gastroenterology, Department of Internal Medicine Good Gang-An Hospital Busan Republic of Korea; 3 Department of Medical Humanities and Social Medicine, College of Medicine Kosin University Busan Republic of Korea

**Keywords:** fatty liver, deep learning, transfer learning, classification, regression, magnetic resonance imaging–proton density fat fraction, multi-view ultrasound images, artificial intelligence, machine imaging, imaging, informatics, fatty liver disease, detection, diagnosis

## Abstract

**Background:**

Fat fraction values obtained from magnetic resonance imaging (MRI) can be used to obtain an accurate diagnosis of fatty liver diseases. However, MRI is expensive and cannot be performed for everyone.

**Objective:**

In this study, we aim to develop multi-view ultrasound image–based convolutional deep learning models to detect fatty liver disease and yield fat fraction values.

**Methods:**

We extracted 90 ultrasound images of the right intercostal view and 90 ultrasound images of the right intercostal view containing the right renal cortex from 39 cases of fatty liver (MRI–proton density fat fraction [MRI–PDFF] ≥ 5%) and 51 normal subjects (MRI–PDFF < 5%), with MRI–PDFF values obtained from Good Gang-An Hospital. We obtained combined liver and kidney-liver (CLKL) images to train the deep learning models and developed classification and regression models based on the VGG19 model to classify fatty liver disease and yield fat fraction values. We employed the data augmentation techniques such as flip and rotation to prevent the deep learning model from overfitting. We determined the deep learning model with performance metrics such as accuracy, sensitivity, specificity, and coefficient of determination (*R*^2^).

**Results:**

In demographic information, all metrics such as age and sex were similar between the two groups—fatty liver disease and normal subjects. In classification, the model trained on CLKL images achieved 80.1% accuracy, 86.2% precision, and 80.5% specificity to detect fatty liver disease. In regression, the predicted fat fraction values of the regression model trained on CLKL images correlated with MRI–PDFF values (*R*^2^=0.633), indicating that the predicted fat fraction values were moderately estimated.

**Conclusions:**

With deep learning techniques and multi-view ultrasound images, it is potentially possible to replace MRI–PDFF values with deep learning predictions for detecting fatty liver disease and estimating fat fraction values.

## Introduction

Fatty liver disease is a disease in which fat accumulates in the liver, leading to more severe diseases, such as liver fibrosis, cirrhosis, and liver cancer [1,2]. Fatty liver disease is divided into alcoholic fatty liver disease caused by alcohol consumption and nonalcoholic fatty liver caused by metabolic diseases such as insulin resistance or abdominal obesity [3,4]. While alcoholic and nonalcoholic fatty liver have different etiologies, distinguishing them is very challenging on the basis of subjective symptoms, blood tests, imaging tests, or even histological tests; so, it usually relies on medical history based on alcohol consumption [5-7]. Recently, the prevalence of nonalcoholic fatty liver disease has reached 30% of the world’s population owing to lifestyle changes, and the disease has been investigated to be highly related to cardiovascular disease and other organ cancers, attracting more attention from medical practitioners. Thus, fatty liver disease is considered a critical issue in the field of health care in today’s society, whereas disease symptoms are not noticeable until the disease progresses to a critical stage. Furthermore, the disease is difficult to detect in an early stage owing to the limitation of diagnostic technology.

As of now, a liver biopsy has been regarded as the gold standard for diagnosing fatty liver disease and assessing the degree of fibrosis owing the fatty liver. However, liver biopsy is rarely performed clinically owing to its invasiveness, which can lead to serious complications. In addition, liver biopsy is limited to represent the entire liver because only a small portion of the liver is extracted. As a noninvasive method, imaging methods have been used to diagnose the fatty liver, including ultrasonography, computed tomography, and magnetic resonance imaging (MRI) of the abdomen. The MRI method consists of MRI–proton density fat fraction (MRI–PDFF) or MR spectroscopy [8-12]. The MRI–PDFF method measures fat fraction values in fatty liver, being computed by the ratio of fat protons to fat and water protons in the liver [13]. MR spectroscopy also measures the degree of fatty liver disease. Except for liver biopsy, MRI has been considered the best method in assessing fatty liver, but it is relatively expensive and cannot be carried out in hospitals without MRI equipment. On the other hand, abdominal ultrasound is the most widely used diagnostic method in clinical practice because it is relatively inexpensive and can be performed in most hospitals. However, abdominal ultrasonography has some disadvantages, such that it is highly dependent on the skill of the person conducting the examination and less sensitive to detecting early-stage fatty liver disease. Recently, several studies have been conducted to overcome the limitations of abdominal ultrasound examination and to objectify or automate fatty liver disease diagnosis through abdominal ultrasound examination [14]. Reddy et al [15] demonstrated that ultrasound images could be used to classify fatty liver diseases in computer-aided diagnosis systems, achieving 90.6% classification accuracy. In this context, we aim to develop a model that can classify fatty liver disease using B-mode ultrasound images, and to develop a regression model that can obtain fat fraction values in fatty liver based on a model architecture with the best classification performance.

Several studies have used deep learning (DL) techniques and ultrasound images to classify fatty liver disease and measure fat fraction values. Zhang et al [16] demonstrated that features of B-mode ultrasonic images can be used in a convolutional neural network (CNN)–based model, achieving 90% accuracy. They showed that unique features obtained from ultrasound images could classify fatty liver disease. Similarly, Lin et al [17] presented a novel quantitative ultrasound technique, and Han et al [18] showed a quantitative raw radiofrequency ultrasound signal method to classify fatty liver disease and measure fat fraction values. They demonstrated that preprocessed data obtained from ultrasound images may facilitate a more comprehensive characterization of fatty liver disease. However, to use preprocessed data obtained from ultrasound images, we must use a specific scanner to provide additional information, making classification using ultrasound images difficult. Therefore, we have developed a DL model that can classify fatty liver disease using liver images and kidney-liver images regardless of ultrasound scanners.

With big data sets, there were several pretrained models showing good classification performance. For example, the VGG19 model won the second prize at the 2014 imagenet large-scale visual recognition competition (ILSVRC) [19]. It had the characteristic of architectural simplicity. In addition, InceptionV3 included the batch normalization method and more layers to improve the model performance, which won the first prize at the 2014 ILSVRC [20]. However, since InceptionV3 had a more complex model architecture, people attempted numerous transfer learning methods using VGG19. Furthermore, Resnet included the skip connection method to improve classification performance using complex model architecture; so, this model won the 2015 ILSVRC [21]. Although several pretrained models including more complex model architecture showed good classification performance, they need more computational sources and time. In our previous study, VGG19 provided the best classification performance in terms of sensitivity and area under curve (AUC) scores [22]. Thus, to train our ultrasound image data set, we selected VGG19 that has a comparatively simple architecture and good classification performance.

In this study, we hypothesize that multi-view ultrasound images and DL technology can effectively classify fatty liver disease and measure fat fraction values. In addition, to validate the effectiveness of using multi-view ultrasound images for classification, we evaluated the DL model’s performance on only liver images or kidney-liver images. We identified the decision-making area using a gradient class activation mapping method. Furthermore, we compared the diagnosis of a radiologist with the diagnostic predictions of the DL model using ultrasound images of fatty liver disease and normal subjects without MRI–PDFF values to demonstrate the difference in the 2 diagnoses.

## Methods

### Ethics Approval

This study was approved by the institutional review board at Good Gang-An Hospital (GGAH 2020-06).

### Study Population

**Figure 1:**
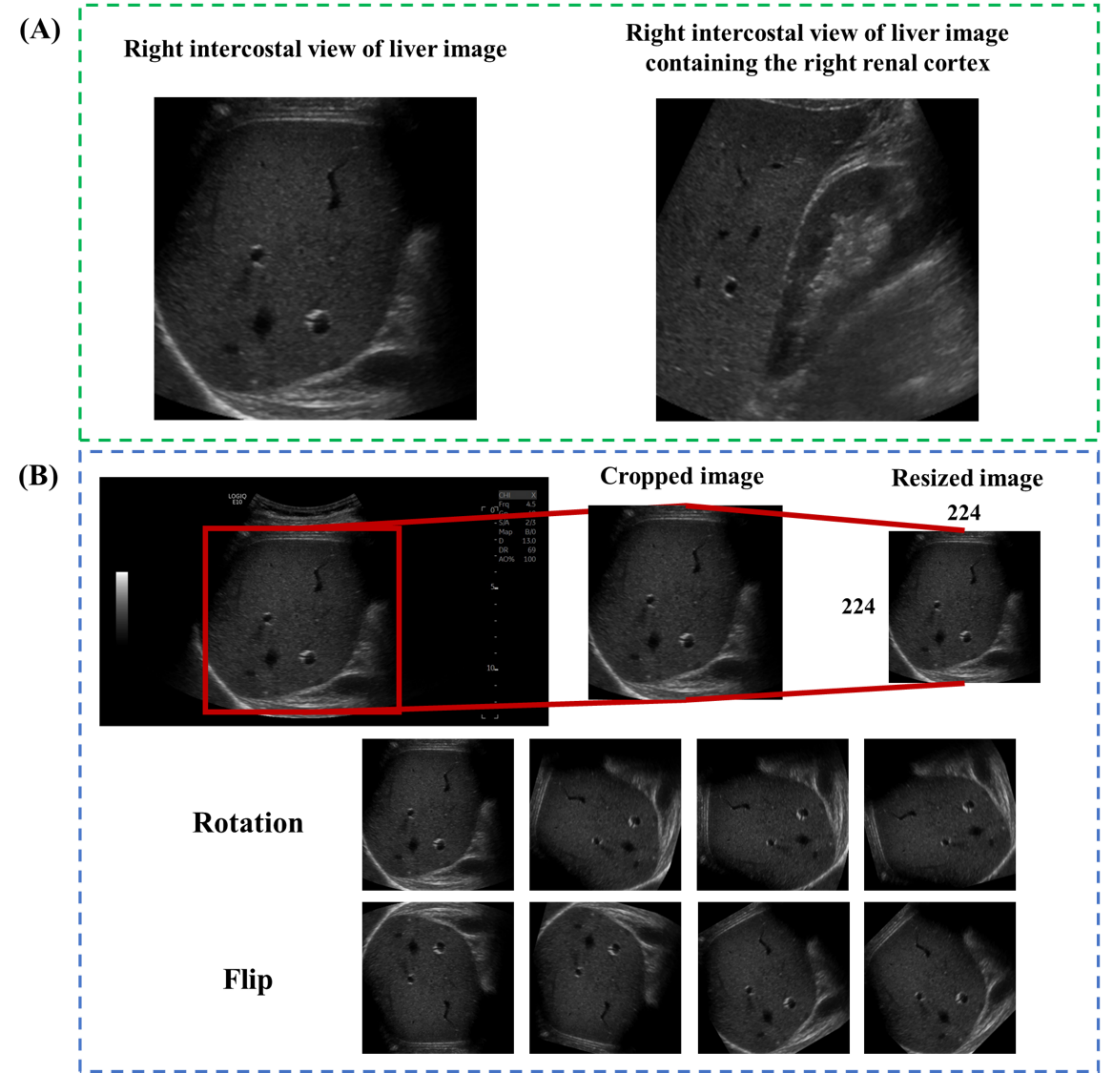
Representative ultrasound images (A) and detailed preprocessing (B).

To classify fatty liver disease, we obtained ultrasound images from 90 subjects with assigned MRI–PDFF values from Good Gang-An Hospital, Busan, Republic of Korea. The subjects comprised 39 individuals with fatty liver disease and 51 normal subjects. The criterion of a 5% MRI–PDFF was used to differentiate subjects with fatty liver from normal subjects [23,24]. From their ultrasound images, we extracted the right intercostal view of the liver (liver image), and the right intercostal view of the liver containing the right renal cortex (kidney-liver image) [14] (Figure 1A). For the DL analysis, we employed 90 liver images and liver-kidney images with MRI–PDFF values, respectively. We further used images of 50 additional subjects without MRI–PDFF values to compare the DL model’s classification performance with the diagnosis of a competent radiologist. Ultrasound images were obtained using either PHILIPS or GE scanners (C5-1/ABD, PHILIPS; LOGIQ E10, GE). In addition, MRI–PDFF values were obtained using either GE or Siemens MR scanners (SIGNA Creator, GE; Skyra, Siemens). The ultrasound images were obtained using 0.5-1 MHz (PHILIPS) and 1-6 MHz (GE) multifrequency transducer. Since PDFF values were obtained in accordance with regions of interest (ROI), we used the average value of PDFF values with ROI. All subjects had ultrasound and MR scans on different days, which varied by an average of 45.1 days. Since clinical test results were collected on the date of recording of MR or ultrasound images, we collected clinical and demographic information obtained on the date of ultrasound imaging. Otherwise, we selected clinical and demographic information recorded as close as possible to the ultrasound imaging date. The metrics of clinical tests included hemoglobin, hematocrit, platelet count, aspartate aminotransferase (AST), alanine aminotransferase (ALT), total bilirubin, albumin, glucose, total cholesterol, high-density lipoprotein (HDL), and low-density lipoprotein (LDL).

### Data Processing

For image preprocessing, the red box region from an ultrasound image was cropped, and the cropped image was resized to a fixed size of 224 × 224 pixels (Figure 1B). To increase the number of ultrasound images, we used data augmentation techniques such as rotation and flipping [25]. We increased the rotation angle by 15° from 0° to 180° to obtain a total of 13 images. In addition, each image was flipped along the x- and y-axes to obtain 39 images from a single image. These image transformation techniques were essential to ensure robustness of the DL model in case samples were not enough. The technique was only applied on training images. To reduce the confounding effect of scanners, the same portions of image samples from respective PHILIPS and GE scanners were provided into the training and testing sets, respectively. In addition, to validate the deep learning model, we employed the 5-fold cross-validation method for all data, which included 17-19 ultrasound images in each fold. Thus, the training images comprised approximately 2800 images for the augmentation method. In addition, we used MinMax scaler to normalize the data to prevent the model from overfitting [26]. The test data were also transformed using this scaler.

### CNN-Based Classification and Regression

**Figure 2:**
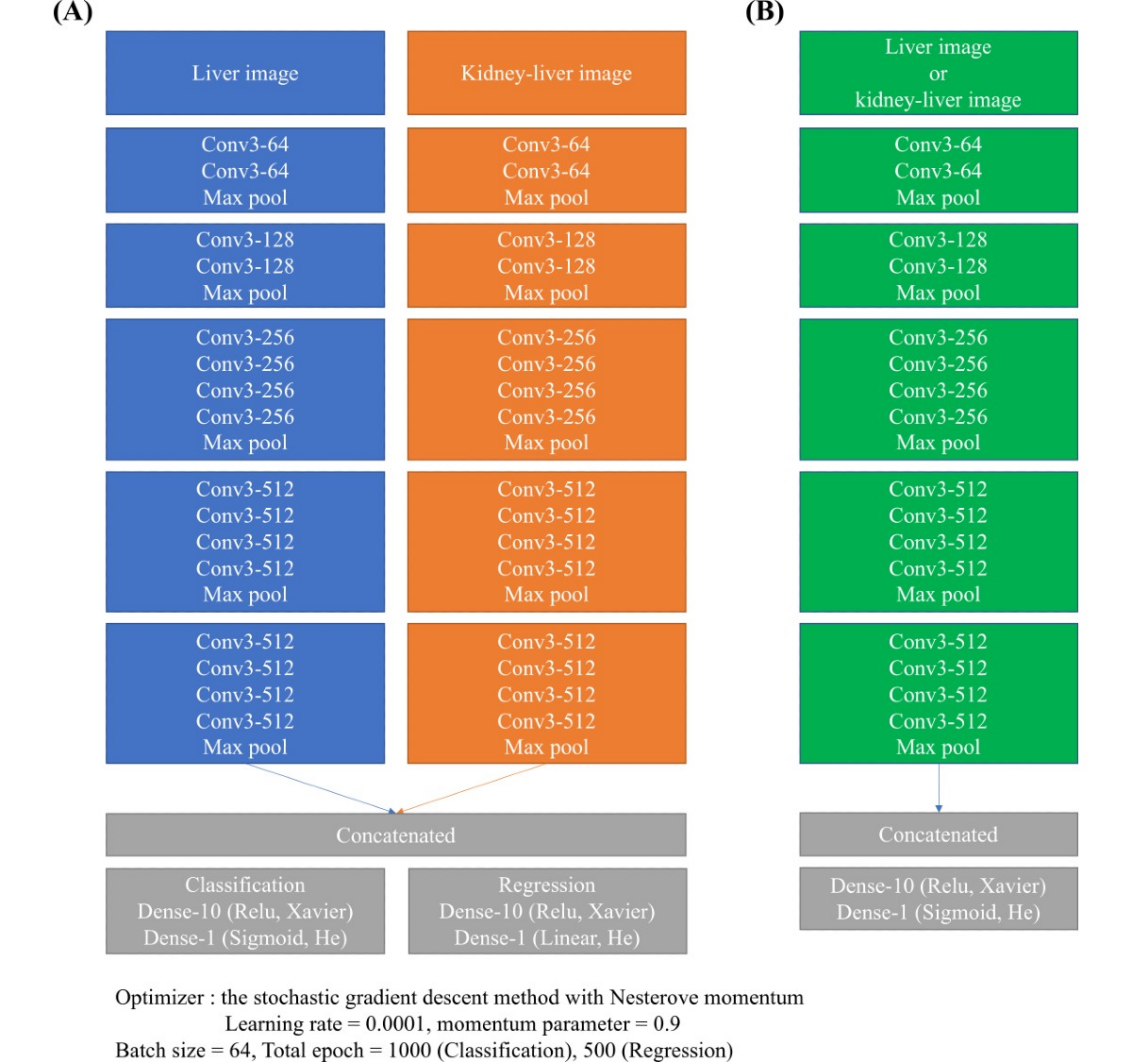
Our model architectures of the combined liver and kidney-liver image model based on 2 VGG19 models (A) and only the liver or kidney-liver image model based on a VGG19 model (B).

Our model architectures of combined liver and kidney-liver (CLKL) images model and only liver or kidney-liver images model are shown in Figure 2. We applied a pretrained DL model of VGG19 on the preprocessed ultrasound images. This model was developed by University of Oxford, being typically used for image classification and localization. We extracted the weights of each node and architecture of the existing model of VGG19. To train the CLKL images, we concatenated their weights at the last layer of each VGG19 model (Figure 2A). In the combined VGG19 model, we constructed 2 layers as the classifier, which were composed of 10 and 1 nodes. We used the Xavier initialization method [27] and the He initialization method [28] in the classifier to improve classification performance. In addition, we used the stochastic gradient descent method with the Nesterov momentum. The momentum parameter is generally used to avoid a local minimum issue because it uses both past and current gradients to update weights in the deep learning model. The momentum parameter has been set to 0.9 in the original VGG19 model; so, we selected the same momentum parameter value [29]. Besides, we selected a learning rate of 10^-4^ because a study [30] using the VGG19 model demonstrated that the learning rate of 10^-4^ showed the best classification accuracy, compared to other learning rates. We also used the sigmoid activation function in the classifier. The regression model was similar to the classification model, but we used the linear activation function in the regressor instead of sigmoid function. To train only liver or kidney-liver images, we used the VGG19 model and same classifier in combined VGG19 model (Figure 2B). In the training phase, we use 64 batch sizes and 1000 epochs for training. We used the gradient-weighted class activation mapping (Grad-CAM) method to visualize the CNN learning process, generating a 2D spatial heatmap of input images that indicate the important regions of CNN predictions [31]. Furthermore, we employed the SHapley Additive exPlanations (SHAP) method to explain the decision evidence of our model [32].

To confirm the classification performance of the combined pretrained model without fine-tuning, we set control models: the combined pretrained model with fine-tuning and without data augmentation. The pretrained model including convolutional layers and fully connected layers is updated by new data set, which is called the fine-tuning process. Without the fine-tuning process, fully connected layers of the pretrained model were only updated by the new data set.

### Performance Evaluation Methods and Statistical Analysis

We evaluated the pretrained DL model’s performance in 5 different cases, using the preprocessed ultrasound images. The pretrained DL model was tested on only liver images, only kidney-liver images, and both liver and kidney-liver images with or without augmentation and fine-tuning. We used the same data set and ultrasound images in each case to compare the classification performance. We used 6 performance metrics to evaluate the classification performance of the model in each case: accuracy, precision, recall (sensitivity), specificity, and F1 score. These metrics were obtained from a confusion matrix, which consists of true positive, true negative, false negative, and false positive. We used the *R*^2^ score to compare the regression model’s performance in this study with that of other studies [33]. For demographic data, we respectively used the Kruskal-Wallis and Fisher exact tests to compare continuous and categorical data between subjects with fatty liver disease and normal subjects in their history of drinking or the lack thereof (Table 1). Keras library (Keras version 2.2.4) were employed to construct deep learning models in the Python framework (version 3.6.5). In addition, statistical analyses were conducted using R software (version 3.6.1).

**Table 1:** Comparisons of demographic metrics between normal subjects and those with fatty liver with regard to their history of drinking or lack thereof.

Characteristics	No history of drinking (n=74)			History of drinking (n=16)		
	Normal subjects (n=42)	Subjects with fatty liver (n=32)	*P* value	Normal subjects (n=9)	Subjects with fatty liver (n=7)	*P* value
Age (years), mean (SD)	57.29 (11.74)	52.47 (13.55)	.19	53.6 (11.8)	61.3 (2.9)	.14
Females, n (%)	22 (52.4)	14 (43.8)	.49	2 (22.2)	2 (28.6)	>.99
Magnetic resonance imaging–proton density fat fraction (%)	2.96 (0.90)	11.82 (8.74)	<.001	3.11 (1.08)	11.49 (5.49)	<.001
Weight (kg), mean (SD)	63.6 (9.0)	70.5 (10.6)	<.05	73.5 (12.8)	73.1 (9.0)	.71
Height (cm), mean (SD)	165.3 (8.1)	164.6 (9.7)	>.99	170.6 (8.0)	169.2 (5.6)	.60
Hemoglobin (g/dL), mean (SD)	13.8 (1.6)	14.7 (1.9)	<.05	13.6 (2.4)	13.3 (1.5)	.60
Hematocrit (%)	41.1 (4.0)	43.3 (5.4)	<.05	40.2 (6.2)	39.4 (5.0)	.96
Platelet count (10^3^/uL), mean (SD)	170.7 (65.0)	204.4 (82.8)	.16	173.6 (75.9)	141.3 (51.7)	.32
Aspartate transaminase (U/L), mean (SD)	43.7 (37.4)	61.0 (74.3)	.24	40.0 (19.7)	82.4 (39.5)	<.05
Alanine transaminase (U/L), mean (SD)	39.7 (54.1)	58.9 (74.0)	<.05	25.3 (16.5)	42.4 (24.6)	.11
Total bilirubin (mg/dL), mean (SD)	1.07 (1.03)	0.83 (0.40)	.44	1.08 (0.54)	2.02 (2.31)	.13
Albumin (g/dL), mean (SD)	4.09 (0.48)	4.30 (0.44)	<.05	4.17 (0.55)	3.47 (1.01)	.19
Glucose (mg/dL), mean (SD)	118.4 (31.8)	132.5 (67.2)	.78	124.6 (26.5)	137.5 (65.8)	.85
Total cholesterol (mg/dL), mean (SD)	172.6 (61.8)	183.5 (57.8)	.63	162.7 (37.3)	135.0 (48.8)	.31
High-density lipoprotein cholesterol (mg/dL), mean (SD)	52.3 (11.8)	49.1 (16.9)	.10	50.2 (16.6)	41.7 (19.0)	.66
Low-density lipoprotein cholesterol (mg/dL), mean (SD)	104.1 (30.0)	113.9 (39.0)	.36	103.0 (47.1)	93.5 (43.1)	.81

## Results

### Demographic Information

Table 1 shows the comparison between subjects with fatty liver disease and normal subjects with respect to their history of drinking or the lack thereof. Regarding both history of drinking and the no history of drinking groups, age, weight, height, and gender were not significantly different between normal and fatty liver groups. Regarding clinical metrics, hemoglobin, hematocrit, ALT, and albumin values were different between the two groups in no history of drinking group, whereas the AST levels of only subjects with fatty liver were higher than those of control subjects in history of drinking group.

### CNN-Based Classification

**Figure 3:**
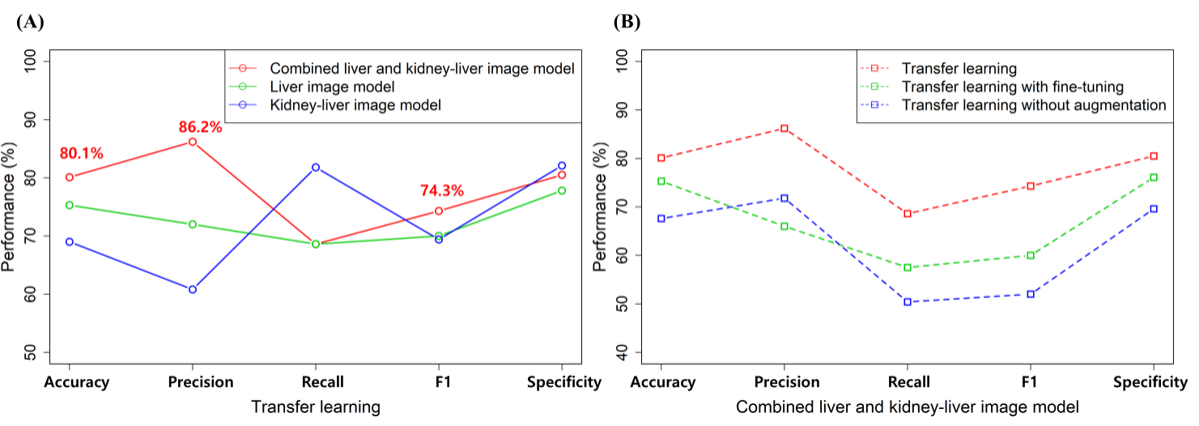
The classification performance of the transfer learning model along with the input ultrasound image view (A) and comparison of classification performance between the transfer learning model and transfer learning with fine-tuning or without augmentation (B), including accuracy, precision, recall, F1 score, and specificity.

**Figure 4:**
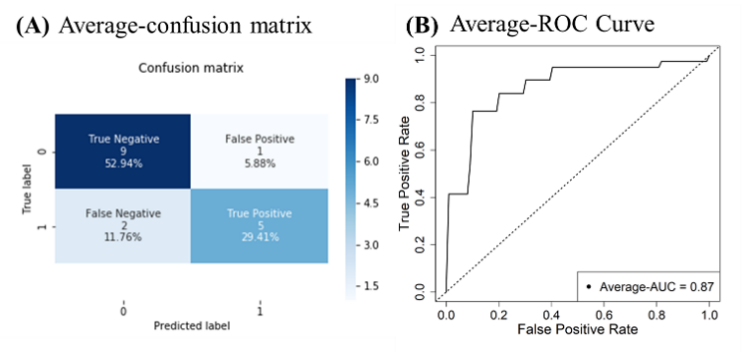
The average-confusion matrix (A) and average-ROC curve (B) using transfer learning model. AUC: area under the curve; ROC: receiver operating characteristic.

**Figure 5:**
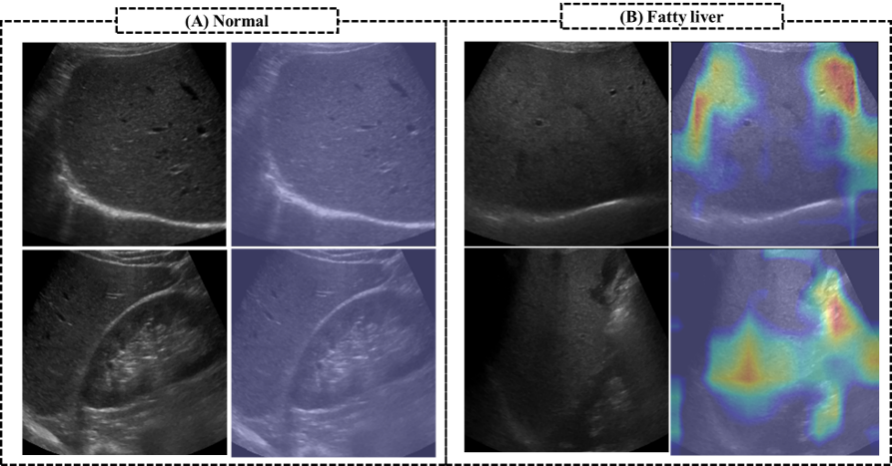
The focal region of CNN predictions of the combined liver and kidney-liver image–trained model. CNN: convolutional neural network.

**Figure 6:**
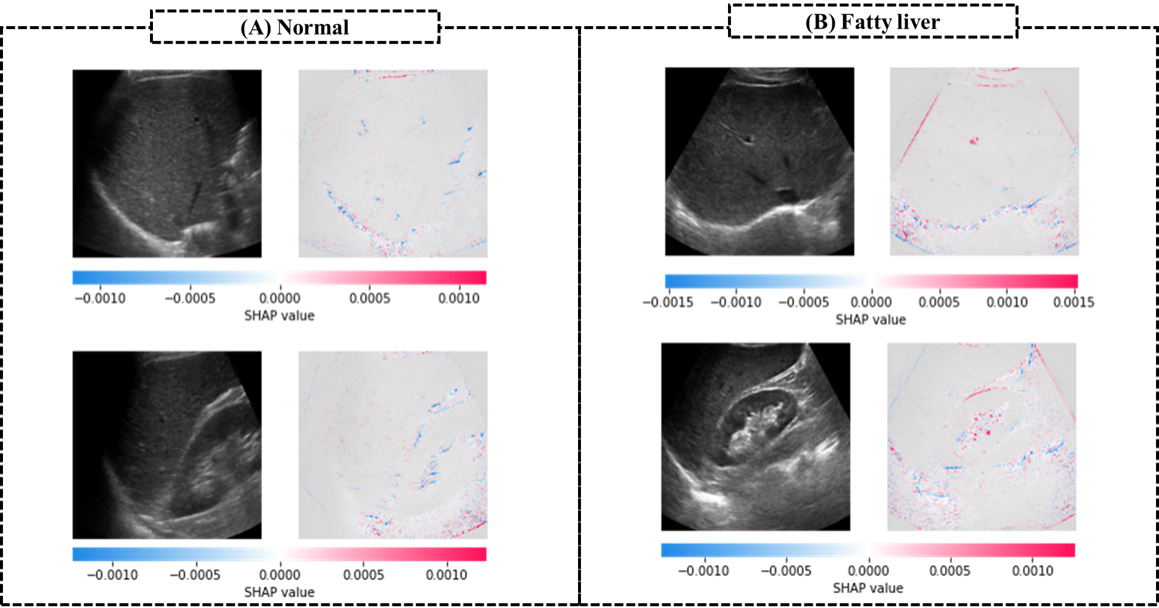
The focal region of CNN predictions of the combined liver and kidney-liver image–trained model using the SHAP method. CNN: convolutional neural network, SHAP: SHapley Additive exPlanations.

Figure 3 shows the accuracy, precision, recall, F1 score, and specificity of the pretrained models along with the types of input image (Figure 3A) and along with transfer learning with or without fine-tuning and without augmentation (Figure 3B). Compared to other models, the CLKL image–trained model had the highest accuracy, precision, and F1 score (Figure 3A). In particular, the precision of the combined model was 86.2%, which is 14.2% higher than that of the other models. The kidney-liver image–trained model had the lowest classification performance with regard to accuracy, precision, and F1 score, whereas the liver image–trained model had the lowest specificity, compared to that of other models. With fine-tuning (Figure 3B), the fine-tuned CLKL image–trained model also had lower accuracy, precision, recall, and F1 scores, compared to those of the transfer learning model without fine-tuning. However, the CLKL image–trained model without fine-tuning had the highest classification performance than that of other models with fine-tuning and without the augmentation method. Figure 4 shows the confusion matrix and the receiver operating characteristic (ROC) curve of the CLKL image–trained model. The CLKL image–trained model had 1 false positive and 2 false negative value in the average-confusion matrix, and the average AUC score was 0.87, indicating that the DL model had good classification performance. To validate the performance of DL model–based predictions, we applied Grad-CAM to the trained CNN model. Figure 5 shows the focal region of CNN predictions of the combined liver and kidney-liver image-trained model using the Grad-CAM method. Figure 5A shows a normal subject’s image, with an MRI–PDFF lower than 5%, and Figure 5B shows an image of a subject with fatty liver, with an MRI–PDFF higher than 5%. The heatmaps highlighted the general liver region in liver images and both the central region of the kidney and liver region in kidney-liver images. Figure 6 shows the focal region of CNN predictions of the CLKL image–trained model using the SHAP method. The SHA*P* values of fatty liver images were positively higher in the hepatic portal and kidney regions. On the other hand, the SHA*P* values of normal images were negatively higher in the liver and kidney regions.

### Regression Model Derived From the Classification Model of the Best Performance

**Figure 7:**
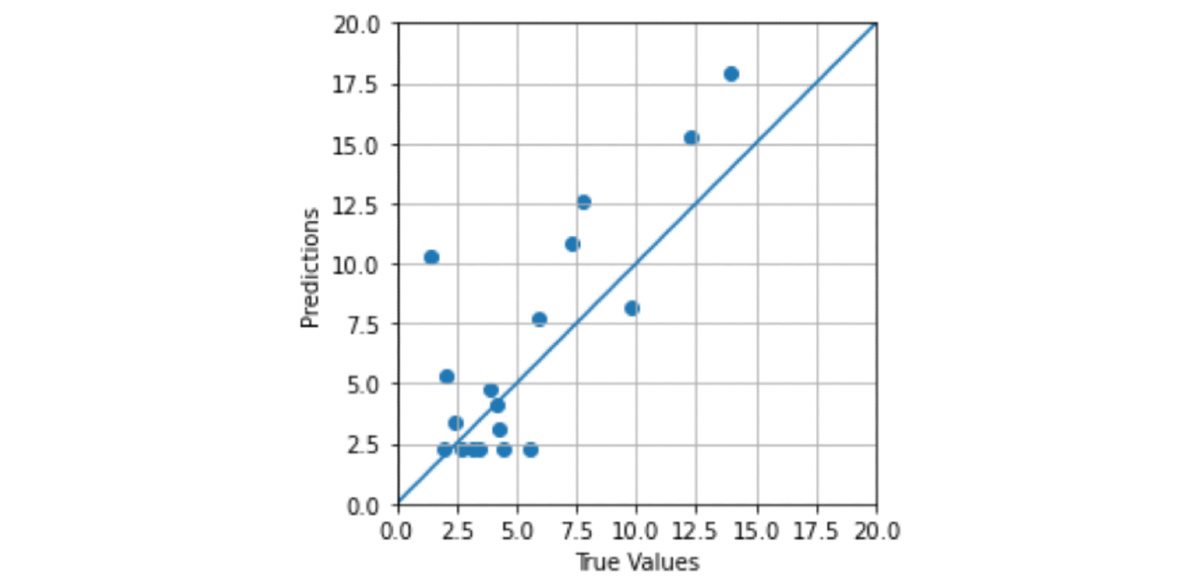
The correlation map of the predicted fat fraction values with MRI–PDFF values. MRI–PDFF: magnetic resonance imaging–proton density fat fraction.

Using the architecture of the CLKL image–trained model, which achieved the best classification performance, we developed the regression DL model using the CLKL images and MRI–PDFF values for 1 among 5 folds. Figure 7 shows the predicted fat fraction values correlated with the MRI–PDFF values, using transfer learning. When training the pretrained DL regression model using the CLKL images, the *R*^2^ score was approximately 0.633, indicating that the predicted fat fraction values were moderately estimated. However, when using 5 folds, the regression models were not trained owing to overfitting problems.

### Comparison Between Radiologist Diagnosis and the CNN-Based DL Model’s Prediction

**Figure 8:**
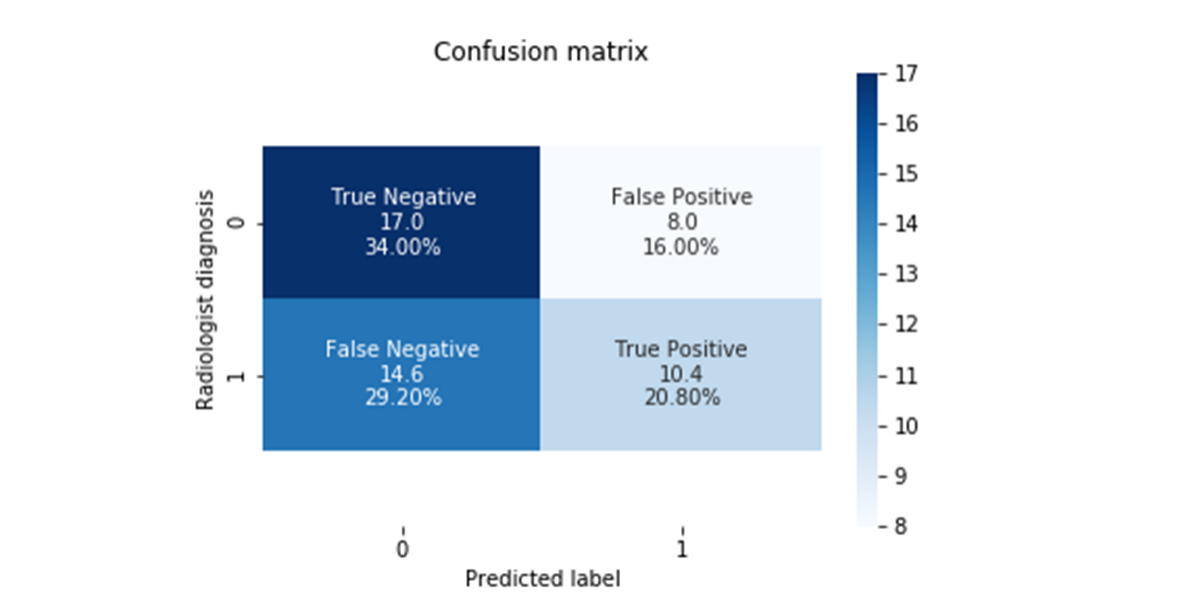
The confusion matrix for the additional subjects without MRI–PDFF values between the pretrained model’s prediction and the radiologist's diagnosis. MRI–PDFF: magnetic resonance imaging–proton density fat fraction.

Using ultrasound images of the additional subjects without MRI–PDFF values, we estimated the predicted classes for the pretrained DL model with the best classification. In addition, we obtained the radiologist’s diagnosis of fatty liver disease for the additional subjects’ ultrasound images and compared it with the model’s prediction. Figure 8 shows the confusion matrix between the classification model and the radiologist’s diagnosis. The accuracy of the pretrained model was 54.8%, which indicates that predictions of the pretrained model were different from the radiologist’s diagnosis.

## Discussion

### Principal Findings

Using the ultrasound images and pretrained DL model, we have demonstrated that multi-view ultrasound images and DL technology could effectively classify fatty liver disease and measure fat fraction values as well, regardless of the disease type, alcoholic or nonalcoholic fatty liver disease. Not only was the classification model’s accuracy 80.1%, but also the *R*^2^ value of the predicted fat fraction values obtained using the regression model was also approximately 0.633. Despite using different scanners to obtain the ultrasound images, the performance of the classification was similar to that of MRI–PDFF values. In addition, to diagnose fatty liver, radiologists often used multi-view ultrasound images including the right intercostal image of the liver and the right intercostal image of the liver including the right renal cortex [14]. We confirmed that the deep learning model could also use those ultrasound images to classify the subjects with fatty liver.

When using a different data set rather than a pretrained data set, transfer learning with fine-tuning originally had better performance than transfer learning without fine-tuning. However, in our study, transfer learning without fine-tuning had better classification performance than that with fine-tuning. This is most likely because the size of our data set for updating the weights of all layers was small. Moreover, for this reason, the regression model also showed poor performance in the 5-fold cross-validation models. On comparing the classification model’s prediction and the radiologist’s diagnosis, the diagnosis and the model prediction for many subjects were inconsistent. However, although the MRI–PDFF values for additional subjects should be confirmed, the possibility that it could be applied clinically in the future could be confirmed by matching for half of the radiologist’s diagnosis (Figure 8).

### Limitations

There were several limitations in this study. First, a radiologist scanned ultrasound images with 2 scanners. As the confounding effect of different scanners may affect DL models, DL models should be developed using ultrasound images scanned from a single scanner. Second, in this study, we collected liver images of subjects with fatty liver disease, including alcoholic fatty liver and nonalcoholic fatty liver disease. However, regardless of the type of fatty liver disease, our model could estimate the predicted fat fraction values and classify fatty liver classes, so this is not a fatal flaw in our study. Third, ultrasound and MR imaging were performed on different dates, which may have a confounding effect on our results. Although our models showed good classification and regression performance, this study has been retrospectively designed using ultrasound images and MRI–PDFF determined on different dates. Thus, ultrasound images and MRI–PDFF obtained on the same date should be used in future studies. Finally, the location of the liver or kidney in the ultrasound images was different; so, this may have a confounding effect on the DL models’ performance. We used a data augmentation technique, including rotation and flip, to reduce the confounding effect of the liver location. Thus, we may be able to free our models from this confounding effect of the liver location.

### Comparison With Prior Work

Several previous studies have used ultrasound images and DL techniques in this context (Table 2). Reddy et al [15] proposed a novel computer-aided diagnosis framework for fatty liver disease. They scanned and collected 86 normal liver images and 76 fatty liver images using the same scanner and used the pretrained DL model with transfer learning and fine-tuning. They obtained 90.6% accuracy, 95% sensitivity, and 85% specificity. Byra et al [34] also proposed a similar DL framework and obtained 96.3% accuracy, 100% sensitivity, and 88.2% specificity using transfer learning and B-mode images scanned using the same scanner. In addition, Han et al [18] proposed a noninvasive diagnosis system of nonalcoholic fatty liver disease and a quantification system of the liver fat fraction values using features extracted from ultrasound images. They collected ultrasound images and MRI–PDFF values of 204 prospectively enrolled participants with nonalcoholic fatty liver disease and participants without fatty liver disease. They used raw radiofrequency ultrasound signals obtained from the ultrasound image scanner and obtained 96% accuracy, 97% sensitivity, 94% specificity, and an R^2^ value of 0.79 using DL techniques. Although the classification performance of our model was inferior to that reported in previous studies, it is inadequate to compare our study with previous studies using the same scanner. It is impossible to generalize the DL model using ultrasound images obtained from the same scanner. Thus, we believe that our study is more generalized than other studies because our study used ultrasound images obtained using 2 different scanners.

**Table 2:** Previously published classification results of the fatty liver versus the normal data sets.

Related work	Data	Methods	Accuracy
Reddy et al [15]	86 normal liver images and 76 fatty liver images using the same scanner	Transfer learning	90.6
Byra et al [34]	B-mode ultrasound images	Transfer learning	96.3
Han et al [18]	Raw radiofrequency ultrasound signals	Convolutional neural network algorithm	96.0
This study	The combined liver and kidney-liver images scanned by 2 scanners (n=90)	Transfer learning	80.1

### Conclusions

In conclusion, using the pretrained DL model and ultrasound images, we demonstrated that transfer learning had the best classification (80.1% accuracy), using multi-view ultrasound images including liver and kidney-liver images. Furthermore, our study demonstrated that the predictions of fatty liver disease using the classification DL models could be implemented in the clinical field without complying with MRI–PDFF values, the gold standard, in the future. A prospective future study is required to develop DL techniques using more ultrasound images with MRI–PDFF values to confirm this study’s results. Future studies can prove that ultrasound images can be used as assistant components in the clinical field, achieving more robust classification and regression performance.

## Abbreviations

ALT: alanine aminotransferase
AST: aspartate aminotransferase
AUC: area under the curve
CLKL: combined liver and kidney-liver
CNN: convolutional neural network
DL: deep learning
Grad-CAM: gradient-weighted class activation mapping
HDL: high-density lipoprotein
ILSVRC: imagenet large-scale visual recognition competition
LDL: low-density lipoprotein
MRI: magnetic resonance imaging
MRI–PDFF: magnetic resonance imaging–proton density fat fraction
ROC: receiver operating characteristic
ROI: regions of interest
SHAP: SHapley Additive exPlanations
